# Combining Calcium Phosphates with Polysaccharides: A Bone-Inspired Material Modulating Monocyte/Macrophage Early Inflammatory Response

**DOI:** 10.3390/ijms19113458

**Published:** 2018-11-03

**Authors:** Hassan Rammal, Camille Bour, Marie Dubus, Laura Entz, Léa Aubert, Sophie C. Gangloff, Sandra Audonnet, Nicolae B. Bercu, Fouzia Boulmedais, Cedric Mauprivez, Halima Kerdjoudj

**Affiliations:** 1 EA 4691, Biomatériaux et Inflammation en Site Osseux (BIOS), SFR CAP Santé (FED4231), Université de Reims Champagne Ardenne, Reims 1100, France; hkrammal@hotmail.com (H.R.); camille.bour@univ-reims.fr (C.B.); marie.dubus@univ-reims.fr (M.D.); l.entz@orange.fr (L.E.); l.aubert@outlook.fr (L.A.); sophie.gangloff@univ-reims.fr (S.C.G.); cedric.mauprivez@univ-reims.fr (C.M.); 2 UFR d’Odontologie, Université de Reims Champagne Ardenne, Reims 51100, France; 3 UFR de Pharmacie, Université de Reims Champagne Ardenne, Reims 51100, France; 4 Plateau Technique URCACyt, Université de Reims Champagne Ardenne, Reims 51100, France; sandra.audonnet@univ-reims.fr; 5 EA 4682, Laboratoire de Recherche en Nanoscience (LRN), Université de Reims Champagne-Ardenne, Reims 51100, France; nicolae-bogdan.bercu@univ-reims.fr; 6 Université de Strasbourg, CNRS, Institut Charles Sadron, UPR22, 23 rue du Loess, BP 84047, 67034 Strasbourg CEDEX 2, France; fouzia.boulmedais@ics-cnrs.unistra.fr; 7 Pôle Médecine bucco-dentaire, Hôpital Maison Blanche, Centre Hospitalier Universitaire de Reims 51100, France

**Keywords:** bone regeneration, inflammation, monocyte, bone inspired material, cytotoxicity

## Abstract

The use of inorganic calcium/phosphate supplemented with biopolymers has drawn lots of attention in bone regenerative medicine. While inflammation is required for bone healing, its exacerbation alters tissue regeneration/implants integration. Inspired by bone composition, a friendly automated spray-assisted system was used to build bioactive and osteoinductive calcium phosphate/chitosan/hyaluronic acid substrate (CaP-CHI-HA). Exposing monocytes to CaP-CHI-HA resulted in a secretion of pro-healing VEGF and TGF-β growth factors, TNF-α, MCP-1, IL-6 and IL-8 pro-inflammatory mediators but also IL-10 anti-inflammatory cytokine along with an inflammatory index below 1.5 (versus 2.5 and 7.5 following CaP and LPS stimulation, respectively). Although CD44 hyaluronic acid receptor seems not to be involved in the inflammatory regulation, results suggest a potential role of chemical composition and calcium release from build-up substrates, in affecting the intracellular expression of a calcium-sensing receptor. Herein, our findings indicate a great potential of CaP-CHI-HA in providing required inflammation-healing balance, favorable for bone healing/regeneration.

## 1. Introduction

A promising approach in tissue repair, healing, and regeneration is the use of biomaterials with bioactive components that control regeneration rate and tissue formation. However, a key feature of the healing process is the generation of an optimal environment with moderate inflammatory signals initiating tissue response, but an exacerbated and/or chronic inflammation may delay and hamper tissue repair [1].

Calcium phosphate (CaP) bioceramics, common inorganic biomaterials, are increasingly used in orthopaedic and dental clinical daily routine. Up to date, a myriad of commercially available bioceramics have been used with mitigated clinical success [2,3]. While most of them are osteoconductive, few are osteoinductive, heavily depending on their solubility. Sintered hydroxyapatite and/or β-tricalcium phosphate (β-TCP) exhibit a very low solubility, which hinders bone remodeling. Room-temperature-formed CaP phases (i.e., calcium-deficient hydroxyapatite, brushite, octacalcium phosphate…) opened up a wide range of possibilities in the manufacturing of new CaP bone substitutes. Advances in biofabrication approaches have recently enabled the buildup of attractive biomaterials that aim to mimic the complex composition of bone. Indeed, the amalgamation of bioceramics with inorganic and organic polymers has drawn lots of attention due to their great potential in regenerative medicine. Several natural polymers including polysaccharides (i.e., pectin, alginate, chitosan, hyaluronic acid derivatives) and proteins (i.e., collagen) have been therefore developed as a base material for polymeric composite scaffolds [4,5,6]. Using a new and straightforward method based on simultaneous spray coating of interacting species process, we successfully designed at mild conditions, a bioactive inorganic/organic substrate made of CaP, chitosan (CHI) and hyaluronic acid (HA) [7,8,9]. Positively charged CHI forms complexes with negatively charged HA, a natural glycosaminoglycan that interacts with cell surface receptors including CD44. The combination of CaP with CHI and HA biopolymers (CaP-CHI-HA) resulted in a bioactive, carbonated and poorly crystalline hydroxyapatite with a composition close to bone mineral phase. Furthermore, CaP-CHI-HA offers interesting properties for bone regenerative medicine as it boosts the early stem cell differentiation into osteoblast-like lineage, maintains stem cell paracrine production of osteoprotegerin, and induces paracrine secretion of angiogenic growth factors such as vascular endothelial growth factor, required for bone vascularization. More interestingly, CaP-CHI-HA provides advantageous functionalities limiting the adhesion of implant-associated *S. aureus*, one of the most serious postoperative complications in orthopedic surgery [8].

Upon bone biomaterial implantation, monocytes reach the implant site interface and differentiate into macrophages with the capacity to secrete an array of pro- and anti-inflammatory cytokines and growth factors directing the biomaterial integration into host bone tissue [10]. However, adverse monocytes/macrophages response can lead to the formation of a fibrous capsule avoiding the biomaterial integration and impairing bone remodeling [11]. With inflammation increasingly recognized as a key component influencing tissue regeneration, unravelling interactions between bio-inspired CaP-CHI-HA and monocytes/macrophages is thought to provide essential information for the rational design of new biomaterial tools. Herein, we aimed to characterize CaP-CHI-HA and CaP in respect to their early inflammatory response and to identify a suitable substrate, which could be favorable for bone regenerative medicine. Thus, we herein aimed to evaluate the influence of amalgamating CaP with CHI and HA on monocytes-like cytotoxicity before assessing their early inflammatory/secretory profile. Finally, the potential implication of both hyaluronic acid receptor (CD44) and calcium sensing receptor (CaSR) in early monocyte-like response were investigated.

## 2. Results and Discussion

Given the importance of macrophages in bone dynamics, many studies have focused on interactions between bone substitute biomaterials and macrophages [12,13], however, immune-regulatory effects of biomaterial surface and chemical composition on monocytes are poorly investigated. Human monocytes THP-1, one of the most established monocyte/macrophage culture models, once activated into macrophages, they adhere to the substrate surface and spread [14]. Adherent THP-1 resembles primary monocyte-derived macrophages in terms of morphology, antigen expression, and secretory products [15]. To overcome limits of primary circulating monocytes (i.e., limited lifespan, low yield, high inter-individual variability), the current study used undifferentiated THP-1 to elucidate the effect of amalgamating polymers as chitosan and hyaluronic acid with calcium phosphate (bio-inspired CaP-CHI-HA) on monocytes activation. Calcium phosphate (CaP), lipopolysaccharides (LPS, 10 ng/mL), a well-known potent stimulator of monocytes, and inert coverslip glass served as internal, positive and negative controls, respectively. Chitosan and hyaluronic acid control substrate, showing an unsuccessful coating of the coverslip glass, was discarded. Finally, to avoid the effect of autocrine activation of THP-1, we determined 24 h as culture study time.

### 2.1. Cytotoxicity Evaluation

**Figure 1:**
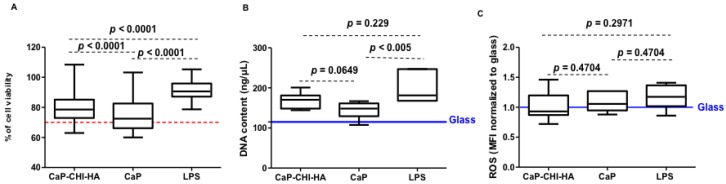
Cytocompatibility. (**A**–**C**): boxes reflecting percentage of cell viability, DNA quantification and intracellular accumulating reactive oxygen species (ROS) normalized to glass control, respectively. Red bar indicates the threshold considered as an indicator of cytotoxic phenomenon, according to ISO standard (ISO/EN 10993 part 5 guidelines) and blue bars indicated DNA content and intracellular accumulating ROS on glass (*n* = 6, Mann Whitney test).

We previously showed that CaP-CHI-HA and CaP are cytocompatible substrates regarding human mesenchymal stem cells [8,9]. When it comes to THP-1, they are considered more sensitive [16]; therefore, the cytocompatibility of CaP-CHI-HA was firstly monitored by WST-1 proliferation assay and DNA quantification. The absorption values of reduced WST-1 reagent normalized to glass negative control are represented in Figure 1A. In contact with CaP-CHI-HA and CaP substrates, THP-1 had a lower mitochondrial activity compared to LPS positive control (*p* < 0.0001, Mann Whitney test), but it remained above the 70% of cell viability threshold, considered as an indicator of cytotoxic phenomenon, according to ISO standard (ISO/EN 10993 part 5 guidelines). DNA quantification, illustrated in Figure 1B, did not show significant variation of measured values for CaP-CHI-HA compared to LPS (*p* = 0.229, Mann Whitney test) but showed a significant decrease for CaP (*p* < 0.005, Mann Whitney test). While optical observations did not reveal differences in THP-1 morphology between conditions (i.e., rounded, clustered and adhered cells highlighted in Figure S1, supplementary section), their low metabolic activities and DNA contents on CaP-CHI-HA and on CaP substrates can be explained by the low density of adhered cells on both surfaces.

Although the specific process by which monocytes adhere onto a biomaterial is not fully understood, a low density of adhered THP-1 seems to be a signature of good biocompatibility of the resulting build-up substrates. This latter was confirmed through the evaluation, by flow cytometry, of intracellular accumulation of reactive oxygen species (ROS) in all seeded THP-1 (rounded, clustered and adhered cells). While oxidative stress plays a central role in the materials toxicity, controlling this stress is one of the effective means of tuning the biological response to materials and improving their biocompatibility [16,17]. No significant effect was observed on the intracellular accumulation of ROS in THP-1 in contact with CaP-CHI-HA (*p* < 0.81, Mann Whitney test), CaP (*p* < 0.81, Mann Whitney test) and LPS (*p* < 0.48, Mann Whitney test) compared to glass (Figure 1C), thus confirming the cytocompatibility of both build-up substrates.

### 2.2. Morphological Investigations:

**Figure 2:**
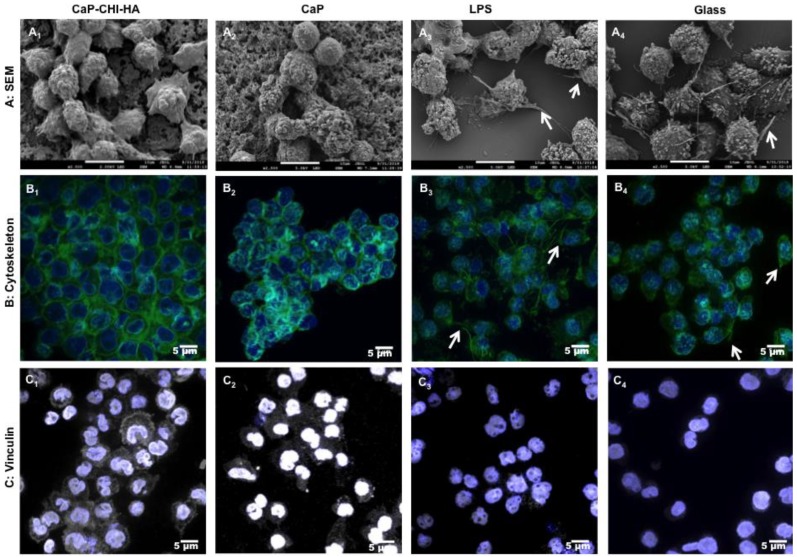
*THP-1 morphology*. Scanning electron microscopy (**A**) views after 24 h of contact with CaP-CHI-HA (**A_1_**), CaP (**A_2_**), stimulated with LPS (**A_3_**) or on glass (**A_4_**), highlighting (i) rounded cell morphology on CaP-CHI-HA and CaP (A_1_ and A_2_, respectively) and (ii) cytoplasmic extensions (arrows) on LPS and glass (A_3_ and A_4_, respectively) (scale bars indicate 10 μm). Confocal laser scanning microscopy (**B**) views of cytoskeleton and (**C**) vinculin after 24h of contact with CaP-CHI-HA (**B_1_** and **C_1_**), CaP (**B_2_** and **C_2_**), stimulated with LPS (**B_3_** and **C_3_**) and on glass (**B_4_** and **C_4_**), showing (i) concentrated F-actin at the membrane with a prominent vinculin distribution throughout the cytoplasm and the membrane in contact with CaP-CHI-HA, CaP (B_1_/B_2_ and C_1_/C_2_, respectively) and (ii) arranged F-actin in spike-like protrusions (arrows) with a peri-nuclear distributed vinculin on LPS and glass (B_3_/B_4_ and C_3_/C_4_, respectively). Green colors correspond to F-actin cytoskeleton, grey to vinculin, blue to nuclei and purple for merged vinculin/nuclei (scale bars indicate 5 μm).

Correlation of cell morphology with surface properties is well established; adhered monocytes/macrophages can exhibit an amoeboid, elongated spindle-like, or rounded shape depending on their lamellipodial extension [10]. To evaluate the morphological response of adhered cells, non-adhered ones were discarded and the remaining THP-1 were followed using scanning electron microscopy (SEM) and confocal laser scanning microscopy (CLSM). While CaP-CHI-HA and CaP showed a heterogeneous population composed by hemispherical THP-1 (Figure 2A_1_,A_2_) with a moderate spread surface area and no developed lamelliopodial extensions, glass and LPS controls revealed the presence of distinct lamelliopodial extensions and an amoeboid shape (Figure 2A_3_,A_4_) as previously described [18]. Furthermore, labelling cell cytoskeleton showed sub-membranous F-actin localization delineating cell boundaries on CaP-CHI-HA and CaP substrates (Figure 2B_1_,B_2_). On glass and LPS controls, in addition to the podosome structure, along with punctuated F-actin on plasma membrane extensions, F-actin was mostly arranged as spike-like protrusions and protruded the cell membrane to form cell motile structures such as lamellipodia and filopodia (Figure 2B_3_,B_4_) [10,19]. Vinculin, linked to focal adhesion complexes, is a key molecule that links the actin cytoskeleton at the membrane. Its recruitment and stabilization to focal adhesion complexes is a signature of a well-established adhesion. On CaP-CHI-HA and CaP substrates, vinculin was more prominent and abundantly distributed throughout the cytoplasm and the membrane (Figure 2C_1_,C_2_), whereas on glass and LPS controls, vinculin was evenly localized at the peri-nuclear region (Figure 2C_3_,C_4_). Based on the absence of cell motile structures and abundant distribution of vinculin in both cytoplasm and membrane, our findings suggest a strong adhesion of THP-1 on the build-up substrates. Biomaterial surface adherent macrophages can fuse to form foreign body giant cells [10], which were not observed in our culture model. Furthermore, it has been proposed that elongation and the presence of plasma membrane extension represent an activated pro-inflammatory (M1) phenotype, whereas a round shape might indicate a resting and non-activated or an anti-inflammatory (M2) phenotype of adhered macrophages [20].

### 2.3. Cytokine Production

**Figure 3:**
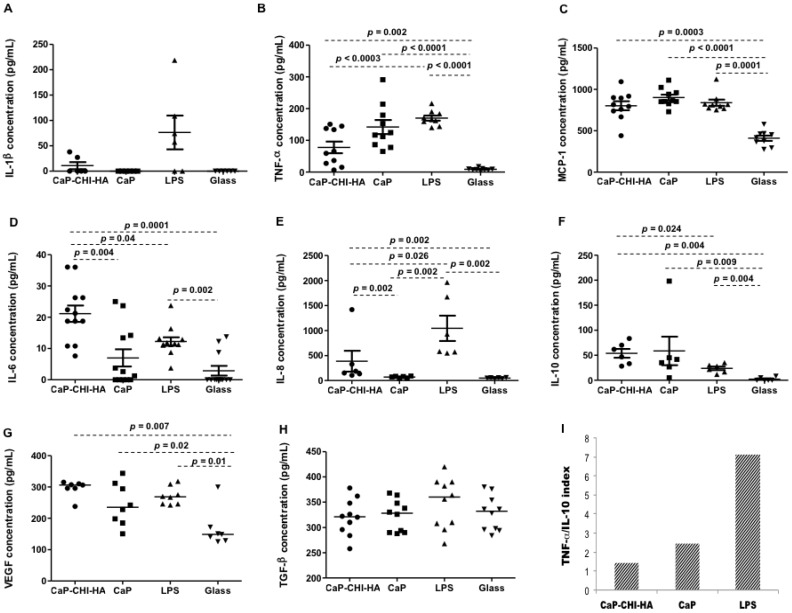
Cytokine, chemokine and growth factors production. Released IL-1β (**A**), TNF-α (**B**), MCP-1 (**C**), IL-6 (**D**), IL-8 (**E**), IL-10 (**F**), VEGF (**G**), TGF-β (**H**) quantified by ELISA, indicating the secretion of pro-inflammatory cytokines/chemokines; concomitant with the secretion of IL-10 anti-inflammatory cytokine by THP-1 in contact with the build-up substrates. Mean TNF-α/mean IL-10 index (**I**), confirming a weak inflammatory profile of THP-1 in contact with the build-up substrates (*n* = 10, Mann Whitney statistical test).

Micro-environmental cues presented by biomaterials (i.e., roughness) are crucial in modulating the immune cell morphology and response [21]. Biomaterial implantation initiates a set of dynamic cellular events that are characterized by distinct pro- and anti-inflammatory derived monocytes recruited to remove necrotic tissue and to begin the healing process through the production of a plethora of mediators [11]. Ambiguity in cytokines is an important parameter during inflammation and healing processes. Pro-inflammatory cytokines, such as interleukin (IL)-1β, and tumor necrosis factor (TNF)-α and chemokines such as monocyte chemo-attractant protein (MCP)-1 are widely expressed at the early stages of implantation, acting as paracrine, autocrine, and potentially endocrine mediators of host response. Furthermore, in vitro studies showed that monocytes/macrophages secrete pro-inflammatory IL-6 and IL-8 in contact with material surfaces [10,22]. IL-6 is mostly described as a pro-inflammatory cytokine, but it is also involved in many regenerative or anti-inflammatory activities [23]. Finally, IL-10, potent anti-inflammatory mediators, vascular endothelial growth factor (VEGF) and transforming growth factor (TGF)-β are produced by monocytes/macrophages and are thought to suppress the biomaterial-induced inflammatory response and contribute to tissue repair, angiogenesis and retain homeostasis [1,10]. Released IL-1β, TNF-α, MCP-1, IL-8, IL-6, IL-10, TGF-β and VEGF by THP-1 in contact with CaP-CHI-HA and controls were thus analyzed by Enzyme-linked immunosorbent assay (ELISA) and results are represented in Figure 3; for a full data description, readers are referred to Supplementary Discussion and figure S2 (supplementary section). As expected, LPS increased the secretion of pro-inflammatory cytokines including TNF-α, MCP-1 and IL-8 with a neglecting increase in IL-10 secretion. Regarding the release of TNF-α pro-inflammatory and IL-10 anti-inflammatory cytokines and the implication of IL-10 in the negative regulation of TNF-α signaling, it is largely described in the literature that the ratio between these cytokines could serve as an index of pro- to anti-inflammatory immune homeostasis [24,25]. Thus, TNF-α/IL-10 index under LPS stimulation was about 7.12, reflecting a high inflammatory environment. Combining CHI and HA biopolymers with CaP, triggered the secretion of pro-inflammatory cytokines/chemokines as TNF–α, MCP-1, IL-6 and IL-8; concomitant with the secretion of IL-10 anti-inflammatory cytokine. The TNF-α/IL-10 index was about 1.45, resulting in a weaker inflammatory profile. Furthermore, CaP-CHI-HA did not exacerbate the release of growth factors as VEGF and TGF-β, known as a powerful activator of angiogenesis and fibrosis, respectively [26]. CaP also influenced the release of TNF–α, MCP-1, IL-6, IL-8, IL-10, VEGF and TGF-β by THP-1 but few differences were noticed compared to CaP-CHI-HA. Indeed, CaP seemed more inflammatory as the concentration of TNF–α was moderately higher with a TNF-α/IL-10 index of about 2.42 (≈ 1.68-fold vs CaP-CHI-HA). However, despite the low values obtained in our conditions, both IL-6 and IL-8 secretions were significantly lower for CaP compared to CaP-CHI-HA (*p* = 0.004 and *p* = 0.002, respectively, Mann Whitney test). These cytokines modulate immune responses and are essential for timely fracture repair with elevated secretion levels during the early phase of bone healing. However, continued and prolonged presence of both cytokines may cause variable degrees of undesired tissue reaction [27].

### 2.4. Are CD44 Receptors Involved in the Early Inflammatory Regulation?

Biomaterial roughness has been shown to influence the inflammatory profile and the macrophage polarization [23,24]. Except the decrease in substrate stiffness [≈2 GPa and 6 GPa for CaP-CHI-HA and CaP, respectively], amalgamation of CaP with biopolymers did not affect the physical properties of the substrate including thickness and roughness [8]. Chitosan and hyaluronic acid biopolymers, depending on their concentration, are described as anti-inflammatory polymers [17]. However, our results suggest that these polysaccharides potentially activate monocytes commitment into a weak inflammatory profile. Although chitosan is not a constituent of the extracellular matrix, most of the biological tissues contain hyaluronic acid, which is a ubiquitously distributed component of the extracellular matrix and in its native form exists as a high molecular weight polymer ranging from 10^5^ to 10^7^ Da [28]. Interaction between the HA receptor (CD44) and the extracellular polysaccharide is connected to a variety of biological functions of monocytes/macrophages, such as adhesion to the extracellular matrix, phagocytosis, migration to inflammatory sites, and secretion of cytokines [19,29]. Thus, we hypothesized that CD44 could be involved in the induction of the weak inflammatory profile in our study design.

**Figure 4:**
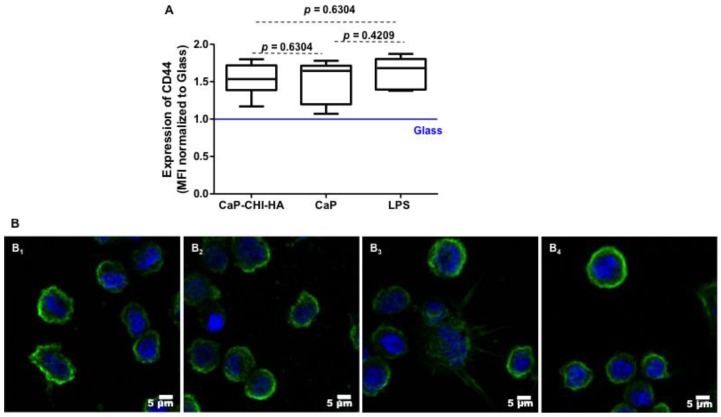
CD44 expression. Median fluorescence intensity normalized to glass, obtained by flow cytometry (*n* = 6, Mann Whitney statistical test) (**A**) and confocal laser scanning microscopy views of CD44 labelled receptors (**B**) expressed by THP-1 in contact with CaP-CHI-HA (**B_1_**), CaP (**B_2_**); stimulated with LPS (**B_3_**) and on glass (**B_4_**) showing clustered and functional receptors at the membrane whatever the studied condition. Green color corresponds to CD44 and blue to nuclei (scale bars indicate 5 μm).

A representative flow cytometry histogram depicting cell-associated fluorescence of THP-1 following probing with CD44, showed a constitutive expression with median fluorescence intensity (MFI) values of about 2143 ± 341 on glass negative control. THP-1 in contact with CaP-CHI-HA and in the presence of the LPS inflammatory environment significantly increased the level of CD44 with MFI values of about 2507 ± 341 and 3535 ± 729 respectively (≈1.16-fold, *p* < 0.01, and ≈1.64-fold, *p* < 0.008, vs glass, Mann Whitney test) and a modest increase in contact with CaP with MFI values of about 3265 ± 802 (≈1.52-fold, vs glass, Mann Whitney test). No statistical difference was observed between CaP-CHI-HA and CaP (Figure 4A). A number of potential mechanisms by which HA exerts its anti-inflammatory properties are suggested [29]. The protective actions of HA are thought to be mediated by its interaction with CD44. However, despite the high expression of CD44 by THP-1 cells, this receptor is in a quiescent state and is unable to bind to HA [30]. Clustering of CD44 on the cell surface has been proposed as a possible mechanism of modulating the HA-binding function of CD44 [7]. Therefore, the functionality of CD44 through its clustering was followed on adhered THP-1 by immuno-fluorescent staining and CLSM visualization. Unexpectedly, our results showed that CD44 was distributed into clusters on the THP-1 membrane whatever the studied condition (Figure 4B). These findings suggested that CD44 on THP-1 is constitutively activated and is not involved in the increase of IL-10 anti-inflammatory cytokine secretion. 

### 2.5. Role of CaSR Receptor in Early Inflammatory Regulation

**Figure 5:**
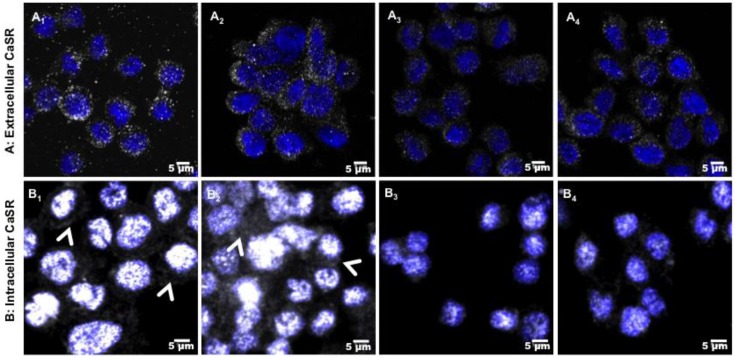
Calcium sensing receptor (CaSR). Fluorescent images of extracellular (**A**) and intracellular (**B**) CaSR by THP-1 in contact with CaP-CHI-HA (**A_1_**,**B_1_**), CaP (**A_2_**,**B_2_**), LPS (**A_3_**,**B_3_**) and glass (**A_4_**,**B_4_**), showing an increase of intracellular labelled CaSR (head arrows) on built-up substrates. Grey colors correspond to CaSR, blue to nuclei and purple for merged CaSR/nuclei (scale bars indicate 5 μm).

CaP-CHI-HA and CaP are both bioactive substrates, inducing carbonated apatite precipitation at their surface and a subsequent decrease in calcium and phosphate concentrations in the extracellular medium. We previously showed that the kinetic release and precipitation of calcium from culture medium onto CaP-CHI-HA was delayed compared to that onto CaP (24 h vs. 4 h, respectively) [8]. An increase in the extracellular calcium concentration was shown to activate monocytes through calcium sensing receptor (CaSR), acting as a mediator of the chemokines/cytokines production [31]. Thus, we hypothesized that CaSR could be involved in the induction of the weak inflammatory profile in our study design. Therefore, CaSR expression and extra/intracellular localization in THP-1 were followed by immuno-fluorescent stainings and CLSM visualizations (Figure 5). Labelled-CaSR was observed on the THP-1 membrane and cytoplasm, indicating the expression of CaSR protein across the plasma membranes and inside the cells. Whatever the studied condition, there was no apparent difference in the expression of membrane CaSR in non-permeabilized cells (Figure 5A). However, a strong fluorescence of labelled-CaSR was observed in THP-1 adhered on CaP-CHI-HA and CaP with both cytoplasmic and perinuclear localisation (Figure 5B_1_,B_2_), whereas on LPS positive and glass negative controls, a weak and punctuated CaSR staining was observed around the nucleus (Figure 5B_3_,B_4_). Thus, our data suggest a possible CaSR implication to sense local changes of Ca^2+^, inducing their intracellular expression.

## 3. Materials and Methods

### 3.1. Substrate Build-Up

An automated spraying device was used for calcium phosphate substrate supplemented with chitosan and hyaluronic acid polymers (CaP-CHI-HA) and calcium phosphate substrate (CaP) build-up, as previously described. For CaP-CHI-HA, CaCl_2_, 2H_2_O (0.32 M), and chitosan (CHI, 75–85% deacetylated, low molecular weight) 0.3 mg/mL were dissolved in NaCl (0.15 M)/HCl (2 mM) buffer (pH 4), whereas NaH_2_PO_4_ (0.19 M) and hyaluronic acid (HA) 0.3 mg/mL were prepared in NaCl (0.15 M) buffer (pH 10). For CaP substrate, a calcium solution of Ca (NO_3_)_4_HPO_4_ (0.32 M) and a phosphate solution of (NH4)_2_HPO_4_ (0.2 M) were prepared in Tris buffer (10 mM Tris, pH 4 and 10, respectively). Briefly, cleaned coverslips (14 mm diameter, Thermo Scientific, Illkirch France were mounted vertically on a mobile holder, on which, both calcium and phosphate solutions were simultaneously sprayed for 2 s followed by a rinsing step of 2 s with ultrapure water and a drying step of 2 s under compressed air.

### 3.2. THP-1 Culture

THP-1 cells, a pro-monocytic cell line, were purchased from the American Type Culture Collection. Cells were cultured at a density of 2 × 10^5^ cells/mL in a 75 cm^2^ flask in RPMI 1640 medium supplemented with 10% heat-inactivated fetal bovine serum, 1% penicillin/streptomycin (*v/v*, Gibco, Villebon-sur-Yvette, France) and maintained in a humidified atmosphere of 5% CO_2_ at 37 °C with a medium change every 2 days. THP-1 were seeded in 24-well plates at 5 × 10^5^ cells/well on UV-decontaminated CaP-CHI-HA and CaP coated coverslips. UV-decontaminated uncoated glass coverslips and lipopolysaccharide treatment (LPS, 10 ng/mL, *E. coli* 0111:B4, Sigma, St. Quentin Fallavier, France were used as negative and positive controls of inflammation. After 24 h of contact, supernatants were collected, centrifuged at 300 *g* and conserved at −20 °C (conditioned media).

#### 3.2.1. Mitochondrial Activity 

WST-1 cell proliferation assay (Roche Diagnostics, Indianapolis, Indiana) was performed on adhered THP-1 on CaP-CHI-HA and on controls for 24 h in accordance with the manufacturer protocol. Absorbance was measured at 440 nm using a FLUOstar Omega microplate reader (BMG Labtech, Mornington, Australia) against a background control as blank. A wavelength of 750 nm was used as the correction wavelength. Mitochondrial activity, an indicator of cell viability, was calculated as the absorbance ratio between sample and glass control (considered as 100% of viable THP-1).

#### 3.2.2. DNA Quantification

DNA of adhered THP-1 on CaP-CHI-HA and on controls for 24 h were extracted using MasterPureTM DNA Purification Kit (Epicentre Biotechnologies, Madison, WI, USA) in accordance with the manufacturer protocol. The quantity of extracted DNA was assessed by measuring the absorbance at 260 nm (Nanodrop, Thermo Scientific, Rockford, IL, USA) with 260/280 nm absorbance ratio for all measured samples comprised between 1.8 and 2.

#### 3.2.3. Reactive Oxygen Species (ROS) Production Analysis

Quantitative measurements of ROS, namely superoxide radicals in THP-1 cells undergoing oxidative stress, were performed using the Muse Oxidative Stress kit (Millipore, Bedford, MA, USA) in accordance with the manufacturer protocol. Briefly, after 24 h of contact with CaP-CHI-HA and controls, adhered and non-adhered THP-1 were re-suspended at a concentration of 1 × 106 cells/mL in 1× assay buffer and incubated in oxidative stress working solution for 30 min at 37 °C. ROS positive cells, determined as median fluorescence intensities (MFI), were assessed by flow cytometry using BD LSRFortessa flow (BD Biosciences, San Jose, CA, USA) cell analyzer and BD FACSDiva (BD Biosciences, San Jose, CA, USA) and FlowLogic (Inivai Technologies, Melbourne, Australia) software.

#### 3.2.4. Scanning Electron Microscopy with a Field Emission Gun (FEG-SEM)

Adhered THP-1 on CaP-CHI-HA and on controls were fixed with 2.5% (*w/v*) glutaraldehyde (Sigma-Aldrich, Saint-Quentin Fallavier, France) at room temperature for 1 h. Samples were dehydrated in graded ethanol solutions from 50 to 100% and desiccated in hexamethyldisilazane (Sigma-Aldrich, Saint-Quentin Fallavier, France) for 10 min. After air-drying at room temperature, samples were sputtered with a thin gold–palladium film under a JEOL ion sputter JFC 1100 and viewed using FEG-SEM (JEOL JSM-7900F, Croissy, France). Images were acquired from secondary electrons at primary beam energy between 5 and 20 kV.

#### 3.2.5. Cytoskeleton Visualization

Adhered THP-1 on CaP-CHI-HA and on controls were fixed with 4% (*w/v*) paraformaldehyde (Sigma-Aldrich, Saint-Quentin Fallavier, France) at 37 °C for 10 min and permeabilized with 0.5% (*v/v*) Triton ×-100 for 5 min. Alexa^®^ Fluor-488 conjugated-Phalloidin^®^ (1/100 dilution in 0.1% Triton ×-100) was used to stain F-actin for 45 min at room temperature. Nuclei were counter-stained with 4,6-diamidino-2-phenylindole (DAPI, 100 ng/mL, 1/10,000 dilution) for 5 min. Stained cells were mounted and imaged by confocal laser scanning microscopy (CLSM, Zeiss LSM 710 NLO, 63× oil immersion objective, Numerical Aperture 1.4, Carl Zeiss Jena, Germany).

#### 3.2.6. Vinculin, CaSR and CD44 Immunolabellings 

Adhered THP-1 on CaP-CHI-HA and on controls were fixed and permeabilized as previously described. After being blocked, THP-1 were incubated for 1 h with mouse polyclonal antibody targeting vinculin (US-Biological, Salem, MA, USA) and rabbit polyclonal antibody targeting CaSR (life technology, Gaithersburg, MD, USA) at a 1/25 and 1/100 dilution in blocking buffer, respectively. Regarding the membrane labelling, fixed THP-1 were incubated for 1 h with rabbit polyclonal antibody targeting CaSR and mouse monoclonal antibody targeting CD44 (Santa Cruz) at a 1/100 and 1/150 dilution in blocking buffer, respectively. After a double DPBS rinsing step, secondary horse anti-mouse and goat anti-rabbit IgG biotinylated antibodies (Invitrogen, Grand Island, NY, USA) were used at a 1/100 dilution for 30 min at room temperature followed by Alexa-488 conjugated streptavidin at 1/2000 dilution (Invitrogen) for 30 min at room temperature. Nuclei were counter-stained with DAPI. Stained cells were finally mounted and imaged by CLSM (Zeiss LSM 710 NLO, 63× oil immersion objective, Numerical Aperture 1.4, Germany).

#### 3.2.7. CD44 Quantification

Adhered and non-adhered THP-1 on CaP-CHI-HA and on controls were re-suspended at a concentration of 1 × 10^6^ cells/mL, labelled and analyzed by flow cytometry (BD LSRFortessa, BD Biosciences, San Jose, CA, USA) through the expression of cluster of differentiation CD44 using FITC-conjugated mouse anti-human CD44-(C26), for 1 h at room temperature, and appropriate isotype controls. Stained cells were immediately analyzed (10,000 events) and performed with the BD FACSDiva (BD Biosciences, San Jose, CA, USA) and FlowLogic (Inivai Technologies, Melbourne, Australia) software.

#### 3.2.8. Cytokine, Chemokine and Growth Factor Releases

The quantification of IL-1β, TNF-α, MCP-1, IL-6, IL-8, IL-10, VEGF and TGF-β proteins in conditioned supernatants was assessed using ELISA MAX^®^ Deluxe Sets for Human IL-6 and IL-8 (BioLegend, San Diego, USA) and DuoSet^®^ ELISA Kit for human TGF-β, TNF-α, IL-10, MCP-1, VEGF and IL-1β (R&D Systems, Lille, France). Absorbance was measured at 450 nm with correction of non-specific background at 570 nm according to the manufacturer’s instructions.

### 3.3. Statistical Analysis

All results were obtained with six independent spray experiments. Results were represented on histograms as mean ± standard error of the mean using GraphPad^®^ Prism 5 software. ELISA results, performed on 10 conditioned media samples, are presented as median. All statistical analysis were performed using GraphPad^®^ Prism 5 software. For Mann Whitney test, a value of *p* < 0.05 was accepted as statistically significant *p* (rejection level of the null-hypothesis of equal medians).

## 4. Conclusions

Herein, we report that build-up substrates provide a cytocompatible and a moderate early inflammatory environment, which could be favorable for bone healing and regeneration. Combining chitosan and hyaluronic acid biopolymers to calcium phosphate mineral provided a fine-tuning of inflammation through the monocyte-like secretion of pro-healing growth factors, either pro- and anti-inflammatory mediators along with an inflammatory index below 1.5 in contrast to CaP with an inflammatory index above 2.5. Although CD44 receptor seems not to be involved in the inflammatory index regulation, our findings suggest a potential role of the chemical composition of the build-up substrates, which might interact with the cell membrane, thus affecting the intracellular expression of CaSR. However, with a similar expression and localization noticed for both bioactive CaP-CHI-HA and CaP substrates, CaSR does not appear to be the one responsible for the over-secretion of IL-6 and IL-8. Other actors sensitive to intra and extracellular calcium modulation such as protease-activated receptor-2 [32,33] could be involved in early monocyte response.

## Supplementary Materials

Supplementary materials can be found at http://www.mdpi.com/1422-0067/19/11/3458/s1.

## Abbreviations

CaP	Calcium Phosphate
CHI	Chitosan
HA	Hyaluronic acid
CaSR	Calcium sensing receptor
CD	Cluster of Differentiation
WST-1	Water-Soluble Tetrazolium Salt-1
ROS	Reactive oxygen species
DNA	Deoxyribonucleic Acid
ELISA	Enzyme-Linked Immunosorbent Assay
FEG-SEM	Scanning electron microscopy with a field emission gun
CLSM	Confocal laser scanning microscopy
