# Supplementary material for: Combining Calcium Phosphates with Polysaccharides: A Bone-Inspired Material Modulating Monocyte/Macrophage Early Inflammatory Response
Source: Int J Mol Sci. 2018 Nov 3;19(11):3458. doi: 10.3390/ijms19113458 (PMC6274876; doi:10.3390/ijms19113458)
Supplement: Supplementary file 1 [file ijms-19-03458-s001.pdf]

International Journal of *Molecular Sciences*

MDPI

Type of the Paper (Article)

Combining Calcium Phosphates with Polysaccharides: A Bone Inspired Material Modulating Monocyte/Macrophage Early Inflammatory Response

Hassan Rammal[1,2], Camille Bour[1], Marie Dubus[1,3], Laura Entz[1], Léa Aubert[1], Sophie C. Gangloff[1,3], Sandra Audonnet[4], Nicolae B. Bercu[5], Fouzia Boulmedais[6], Cédric Mauprivez[1,2,7], Halima Kerdjoudj[1,2]**

[1] EA 4691, Biomatériaux et Inflammation en Site Osseux (BIOS), SFR CAP Santé (FED4231), Université de Reims Champagne Ardenne, Reims, France ; hkrammal@hotmail.com (H.R); camille.bour@univ-reims.fr (C.B); marie.dubus@univ-reims.fr (M.D); l.entz@orange.fr (L.E); l.aubert@outlook.fr (L.A); sophie.gangloff@univ-reims.fr (S.C.G); cedric.mauprivez@univ-reims.fr (C.M); halima.kerdjoudj@univ-reims.fr (H.K)

[2] UFR d'Odontologie, Université de Reims Champagne Ardenne, Reims, France

[3] UFR de Pharmacie, Université de Reims Champagne Ardenne, Reims, France

[4] Plateau technique URCACyt, Université de Reims Champagne Ardenne, Reims, France ; sandra.audonnet@univ-reims.fr (S.A)

[5] EA 4682, Laboratoire de Recherche en Nanoscience (LRN), Université de Reims Champagne-Ardenne, Reims, France ; nicolae-bogdan.bercu@univ-reims.fr (N.B)

[6] Université de Strasbourg, CNRS, Institut Charles Sadron, UPR22, 23 rue du Loess, BP 84047, 67034, Strasbourg Cedex 2, France ; fouzia.boulmedais@ics-cnrs.unistra.fr (F.B)

[7] Pôle Médecine bucco-dentaire, Hôpital Maison Blanche, Centre Hospitalier Universitaire de Reims, France

* Correspondence: halima.kerdjoudj@univ-reims.fr ; Tel: (+33) 3 26 91 80 12

Received: date; Accepted: date; Published: date

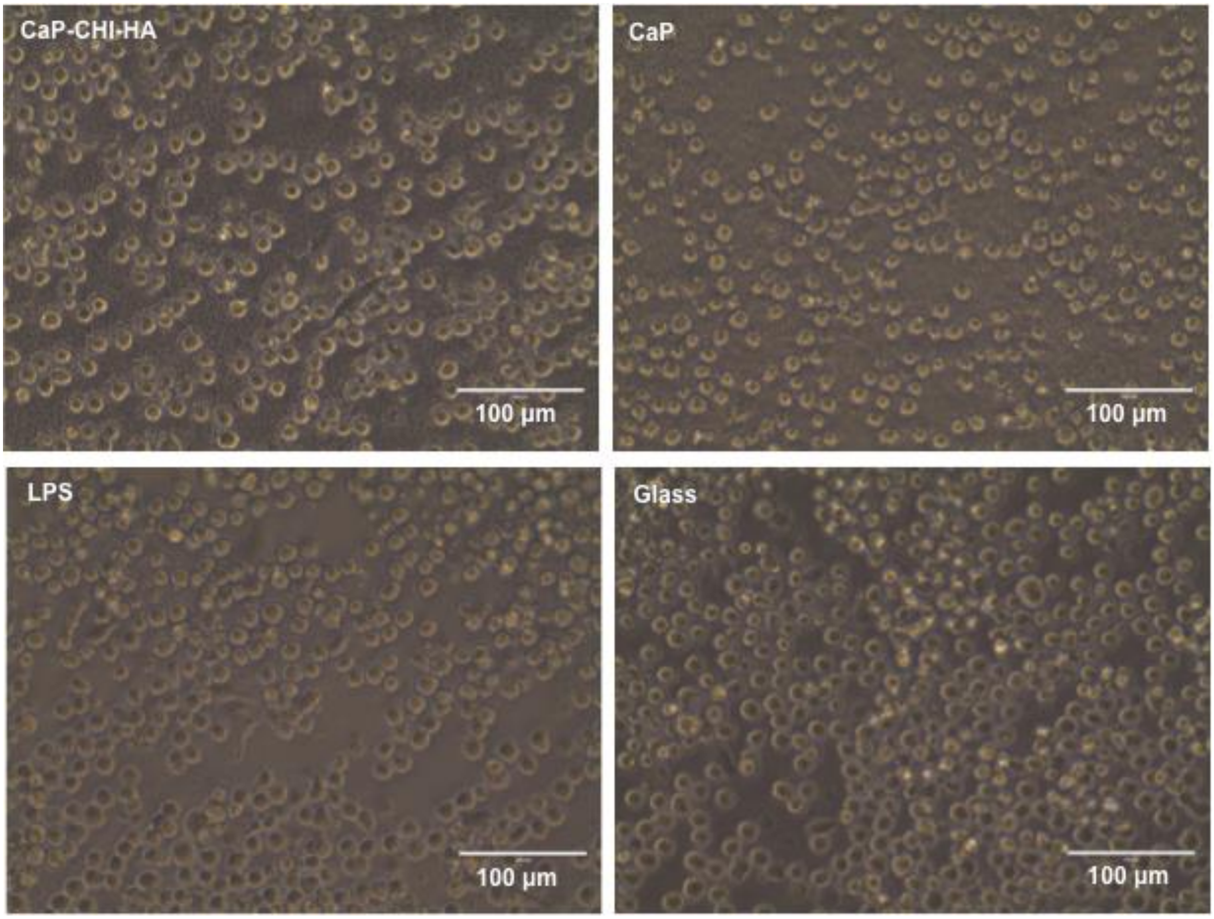

Figure SI-1: *Morphology of THP-1.* Optical microscopy observations of THP-1 in contact with CaP-CHI-HA substrate. CaP substrate, LPS and inert coverslip glass served as internal, positive and negative controls, respectively, showing rounded, clustered and adhered cells whatever the condition (scale bar indicates 100 µm).

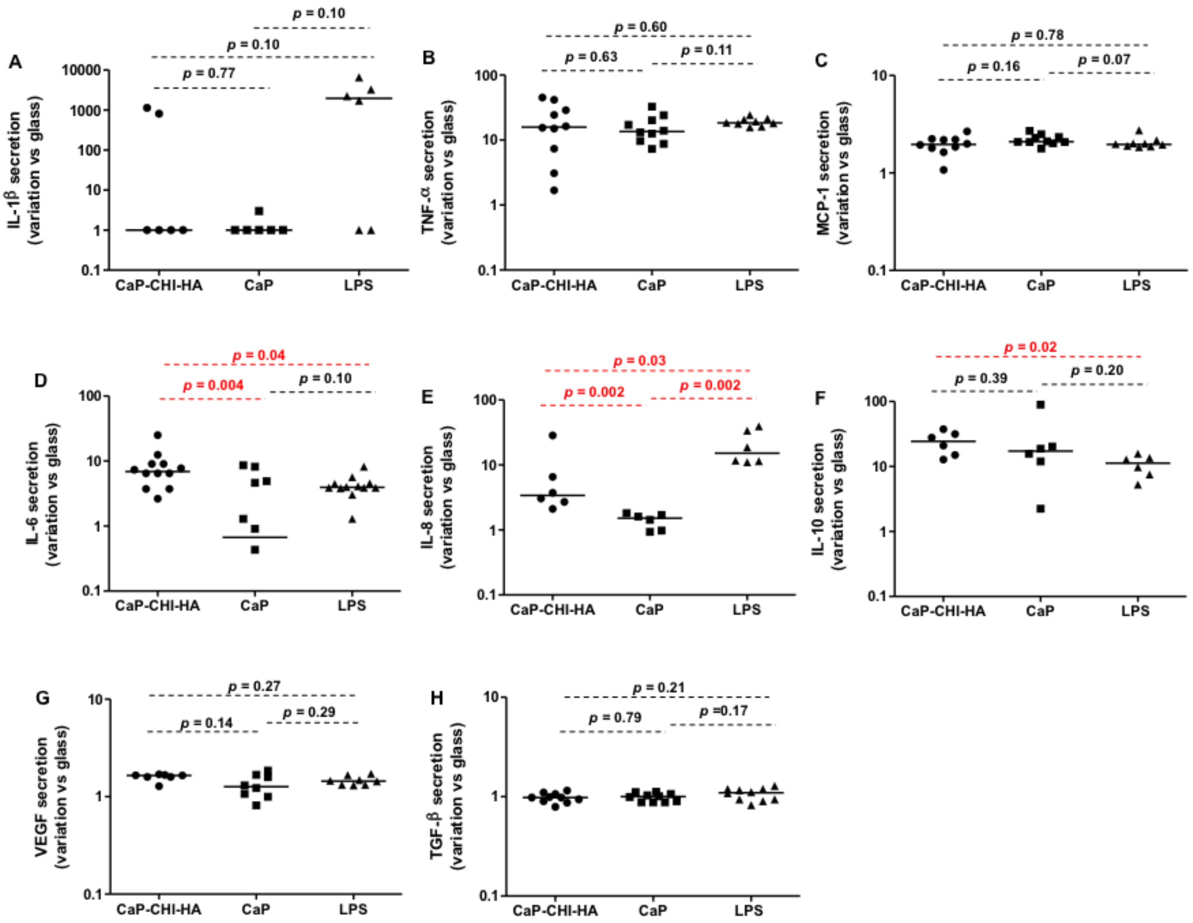

Figure SI-2: *Cytokine, chemokine and growth factor production*. Released TNF-α (A), IL-1β (B), MCP-1 (C), IL-6 (D), IL-8 (E), IL-10 (F), VEGF (G), TGF-β (H) quantified by ELISA and normalized to glass negative control, indicating a significant increase of IL-6 and IL-10 and a significant decrease of IL-8 in contact with CaP-CHI-HA compared to LPS inflammatory control.

Additional discussion:

Monocytes and macrophages are central cells of innate immunity system, playing a key role in inflammation and wound healing and producing a plethora of mediators upon inflammatory activation [1]. Released IL-1β, TNF-α, MCP-1 by THP-1 in contact with CaP-CHI-HA was firstly analysed by Enzyme-linked immunosorbent assay (ELISA). The pro-inflammatory IL-1β, involved in the foreign body immune response was below the detection limit of ELISA kit (4 pg/mL) for CaP and glass [1]. In contrast, a slight increase of IL-1β was noticed in THP-1 supernatants in contact with CaP-CHI-HA and for LPS (10 pg/mL, p=0.922 for CaP-CHI-HA and 76 pg/mL, p=0.103 for LPS *vs* glass, Mann Whitney test) (Figure 3A and SI-2A). THP-1 on CaP-CHI-HA and CaP as well as in presence of LPS, increased significantly protein levels of TNF-α (≈ 8 to 19-fold, p<0.0001, *vs* glass, Mann Whitney test) and MCP-1 (≈ 2-fold, p<0.0001, *vs glass*, Mann Whitney test) in supernatants (Figure 3B and 3C). Furthermore, we distinguished a significant decrease in TNF-α release in contact with CaP-CHI-HA *vs* LPS (p<0.0003, Mann Whitney test), while no statistical differences in MCP-1 release were observed in CaP-CHI-HA and CaP *vs* LPS (Figure SI-2B and SI-2C). Related to CaP, a slight decrease in TNF-α content was noticed (p=0.11, *vs* LPS, Mann Whitney test) and (p=0.063, *vs* CaP-CHI-HA, Mann Whitney test). During bone healing, the effect of IL-1β overlaps with that of TNF-α, contributing to the reparative phase either directly by affecting endothelial cells, osteoclasts and osteoblasts activities or indirectly by inducing additional cytokines and growth factors secretion, and are thus of a potential importance in implant osseo-integration [2]. Furthermore, MCP-1 chemokine has a critical role in macrophage recruitment, immune-regulatory and inflammatory processes involved in tissue repair [3]. The effect of material surfaces on cytokine secretion has been predominately studied *in vitro* [3], and it was reported that monocytes/macrophages secrete pro-inflammatory IL-6 and IL-8 as well as anti-inflammatory IL-10 in contact with material surfaces [4]. However, these secretions were generally low in absence of any exogenous stimulus. Despite the low values obtained in our conditions, still above the detection limit of ELISA kit (6 pg/mL), we noticed a significant increase in IL-6 in contact with CaP-CHI-HA (≈ 8-fold, p<0.0001, *vs* glass, Mann Whitney test) and in presence of LPS (≈ 4-fold, p<0.002, *vs* glass, Mann Whitney test) but a moderate increase in contact with CaP (≈ 2-fold, p=0.12, *versus* glass, Mann Whitney test) (Figure 3D). Compared to LPS inflammatory environment, IL-6 secretion was significantly increased in contact with CaP-CHI-HA (≈ 2-fold, p=0.042, Mann

Whitney test) but moderately decreased in contact with CaP ($p=0.10$, Mann Whitney test). Additionally, IL-6 secretion level was significantly increased in CaP-CHI-HA compared to CaP (≈ 3-fold, $p=0.004$, Mann Whitney test) (Figure SI-2D). Regarding IL-8 secretion, we observed a significant increase in contact with CaP-CHI-HA (≈ 7-fold, $p<0.002$, *vs* glass, Mann Whitney test) and in presence of LPS (≈ 20-fold, $p<0.002$, *vs* glass, Mann Whitney test) and a comparable concentration in contact with CaP and glass ($p=0.13$, *vs* glass, Mann Whitney test) (Figure 3E). Compared to LPS inflammatory environment, IL-8 secretion was significantly decreased in contact with both CaP-CHI-HA and CaP ($p=0.026$ and $p=0.002$, respectively, Mann Whitney test). Moreover, in contact with CaP-CHI-HA, THP-1 increased significantly the secretion of IL-8 compared to CaP (≈ 5-fold, $p=0.002$, Mann Whitney test) (Figure SI-2E). Biomaterial implantation initiates a set of dynamic cellular events that are characterized by distinct pro- and anti- inflammatory cells recruited to remove necrotic tissue and to begin the healing process [5,6]. Ambiguity of cytokines is also an important factor during inflammation and healing process. IL-6 is mostly described as a pro-inflammatory cytokine, but it is also involved in many regenerative or anti-inflammatory activities [7]. IL-10, potent anti-inflammatory mediators, TGF-β and VEGF are produced by monocytes/macrophages and are thought to suppress the biomaterial induced inflammatory response and contribute to tissue repair, angiogenesis and retain homeostasis [2,3]. We noticed a significant increase in IL-10 in contact with CaP-CHI-HA (≈ 24-fold, $p<0.004$, *vs* glass, Mann Whitney test) and CaP (≈ 26-fold, $p<0.0009$, *vs* glass, Mann Whitney test) but in presence of LPS, the resulting increase (≈ 23-fold, $p<0.004$, *vs* glass, Mann Whitney test) was below the detection limit of the ELISA kit (30 pg/mL) (Figure 3F). Compared to LPS induced inflammatory environment, IL-10 secretion was significantly increased in contact with CaP-CHI-HA (≈ 3-fold, $p=0.02$, Mann Whitney test) but moderately increased in contact with CaP (≈ 3-fold, $p=0.20$, Mann Whitney test). A comparable concentration was observed in contact with CaP-CHI-HA and CaP ($p=0.4$, Mann Whitney test) (Figure SI-2F). Secretion of VEGF was significantly increased in contact with CaP-CHI-HA, CaP and LPS (≈ 1.50-fold, $p<0.02$, *vs* glass, Mann Whitney test) (Figure 3G), while a comparable concentration of released VEGF was detected in THP-1 supernatants in contact with CaP-CHI-HA, CaP and LPS (Figure SI-2G). Finally, TGF-β secretion by THP-1 in contact with CaP-CHI-HA did not increase compared to controls (Figure 3H and Figure SI-2H). TGF-β is a powerful activator of connective tissue synthesis and fibroblast proliferation contributing then to fibrosis process [8]. As LPS has negligible effect on fibrosis [1], the absence of an

increase of TGF-β in presence of CaP-CHI-HA might reflect a lack of inflammatory fibrosis induction.

References:

1. Jones, K. S. Effects of biomaterial-induced inflammation on fibrosis and rejection. *Semin. Immunol* **2008**, *20*, 130-136. DOI: 10.1016/j.smim.2007.11.005
2. Mountziaris, P. M.; Mikos, A. G. Modulation of the Inflammatory Response for Enhanced Bone Tissue Regeneration. *Tissue Eng. Part B Rev* **2008**, *14*, 179-186. DOI: 10.1089/ten.teb.2008.0038
3. Anderson, J. M.; Rodriguez, A.; Chang, D. T. Foreign body reaction to biomaterials. *Semin. Immunol* **2008**, *20*, 86-100. DOI: 10.1016/j.smim.2007.11.004
4. Jones, J. A.; Chang, D. T.; Meyerson, H.; Colton, E.; Kwon, I. K.; Matsuda, T.; Anderson, J. M. Proteomic analysis and quantification of cytokines and chemokines from biomaterial surface-adherent macrophages and foreign body giant cells. *J. Biomed. Mater. Res. A* **2007**, *83*, 585-596. DOI: 10.1002/jbm.a.31221
5. Dai, X.; Wei, Y.; Zhang, X.; Meng, S.; Mo, X.; Liu, X.; Deng, X.; Zhang, L.; Deng X. Attenuating Immune Response of Macrophage by Enhancing Hydrophilicity of Ti Surface, *Journal of Nanomaterials* **2015**, *2015*, 1-8. DOI: 10.1155/2015/712810
6. Gardner, A. B.; Lee, S. K. C.; Woods, E. C.; Acharya, A. P. Biomaterials-Based Modulation of the Immune System. *BioMed Res. Int* **2013**, *2013*, 1-7. DOI: 10.1155/2013/732182
7. Sugimoto, M. A.; Sousa, L. P.; Pinho, V.; Perretti, M.; Teixeira, M. M. Resolution of Inflammation: What Controls Its Onset? *Front. Immunol* **2016**, *7*, 160. DOI: 10.3389/fimmu.2016.00160
8. Moghaddam, A. S.; Mohammadian, S.; Vazini, H.; Taghadosi, M.; Esmaeili, S. A.; Mardani, F.; Seifi, B.; Mohammadi, A.; Afshari, J. T.; Sahebkar, A. Macrophage plasticity, polarization and function in health and disease. *J. Cell. Physiol* **2018**, *233*, 6425-6440. DOI: 10.1002/jcp.26429
